# Assessing the role of PEKK implant material on cytotoxicity, inflammatory response, and molecular interactions with pro-inflammatory cytokines: An in-vitro and in-silico study

**DOI:** 10.1016/j.jobcr.2025.08.004

**Published:** 2025-08-09

**Authors:** Amrutha Shenoy, Subhabrata Maiti, Selvaraj Jayaram, Pradeep kumar yadalam, Jessy Paulraj

**Affiliations:** a Department of Prosthodontics, Saveetha Dental College and Hospitals, Saveetha Institute of Medical and Technical Sciences, Saveetha University, Chennai, Tamil Nadu, India; b Department of Biochemistry, Saveetha Dental College & Hospitals, Saveetha Institute of Medical and Technical Sciences, Saveetha University, Chennai, Tamil Nadu, India; c Department of Periodontics, Saveetha Dental College and Hospital, Saveetha Institute of Medical and Technical Sciences, Saveetha University, Chennai, Tamil Nadu, India; d Department of Pedodontics, Saveetha Dental College and Hospital, Saveetha Institute of Medical and Technical Sciences, Saveetha University, Chennai, Tamil Nadu, India

**Keywords:** PAEK(Polyaryletherketone), PEEK(Polyetheretherketone), PEKK(Polyetherketoneketone), Cytotoxicity, Molecular docking

## Abstract

**Introduction:**

and aim: Due to its excellent mechanical strength and biocompatibility, Polyetherketoneketone (PEKK) is emerging as a potential substitute for titanium in dental implant applications. The aim of the study was to evaluate its cytotoxicity, pro-inflammatory responses, and molecular interactions to assess its potential in implant applications.

**Methods:**

This study evaluated the cytotoxicity, pro-inflammatory cytokine responses, and molecular interactions of PEKK compared to titanium. Disc-shaped samples (10 mm × 2 mm) were fabricated for each material following ISO standards. Human periodontal fibroblast cells were cultured and treated with the samples for cytotoxicity assessment using the MTT assay, while pro-inflammatory cytokine gene expression (IL-1β, TNF-α) was analyzed via real-time PCR. Molecular docking was conducted using AutoDock to investigate PEKK's binding interactions with cytokines, and data was analyzed with one-way ANOVA and post hoc test (P < 0.05).

**Results:**

PEKK showed comparable cytocompatibility to titanium, yielding similar outcomes in cell viability (P > 0.05) or pro-inflammatory cytokine expression (P > 0.05). Molecular docking revealed strong interactions with IL-1β (−8.9 kcal/mol) and TNF-α (−7.3 kcal/mol).

**Conclusion:**

This study demonstrates that PEKK exhibits comparable cytocompatibility and pro-inflammatory responses to titanium, with a potential to modulate inflammatory pathways. Further in vivo studies are needed to confirm its clinical viability as an implant material.

**Clinical relevance:**

This study gives the clue of PEKK as an aesthetic implant biomaterial and it can be useful as an alternative to Titanium dental implant.

## 1 Introduction

Dental implants have revolutionized modern dentistry, providing effective solutions for tooth replacement.^1^ Titanium implants are traditionally preferred for their excellent biocompatibility and durability[2, 3, 4^]^. However, concerns regarding aesthetic appeal in anterior regions, potential allergic reactions, galvanic corrosion, and MRI artifacts have prompted the search for alternative materials.^5^, ^6^, ^7^ Zirconia-based implants offer enhanced aesthetics and reduced allergic reactions, but their brittleness limits their suitability for posterior applications.^8^^,^^9^ As a result, there has been increasing interest in ultra-high-performance polymeric materials, particularly polyaryletherketones (PAEKs), which are recognized for their excellent mechanical properties, chemical resistance, and biocompatibility.^10^

Among PAEKs, polyetheretherketone (PEEK) and polyetherketoneketone (PEKK) have shown promise as dental implant materials.^10^^,^^11^ PEEK is a durable thermoplastic material characterized by its semi-crystalline structure and high mechanical strength, stiffness, and toughness, along with favorable radiolucency for post-implantation imaging^12^. PEKK shares similarities with PEEK but offers superior mechanical properties, such as higher tensile strength, flexural modulus, and impact resistance.^13^, ^14^, ^15^ These attributes make PEKK particularly suitable for load-bearing applications, while its shock-absorbing capabilities further enhance its appeal in dental implantology. Recent studies suggest that PEKK's stiffness and Young's modulus resemble those of human bone, improving its potential as a dental implant material compared to PEEK.^16^

The successful integration and longevity of dental implants rely on the formation of a strong soft tissue barrier, primarily composed of fibroblasts and keratinocytes. This barrier helps protect the implant and minimize complications such as peri-implant mucositis. Human gingival fibroblasts play a key role in establishing a secure tissue seal and maintaining the extracellular matrix necessary for tissue regeneration and healing. Given the close interaction between implants and host tissues, it is crucial to understand the inflammatory response and cytotoxicity of implant materials.^10^ Adverse effects from these materials can hinder healing or lead to implant failure, making the evaluation of their biological responses essential for assessing their safety and efficacy.

PEKK is increasingly being explored as a viable alternative for implant abutment fabrication, offering stability, comfort, and potential resistance to biofilm formation, its biological response—particularly its effects on oral cells and fibroblasts—remains underexplored.^17^ This study aimed to analyze the cytotoxicity, pro-inflammatory cytokine responses, and gene expression induced by PEKK in oral cells and fibroblasts. Additionally, molecular docking was to be conducted to investigate PEKK's interactions with key pro-inflammatory cytokines, providing insights into its potential role in modulating inflammatory pathways. By comparing PEKK's biological effects to those of traditional titanium implants, the study aimed to determine whether PEKK exhibits comparable responses, supporting its potential as an alternative material in dental implant technology.

## 2 Materials and methods

### 2.1 Study design

This in vitro study assessed PEKK's cytotoxicity, inflammatory response, and gene expression compared to titanium using human cell lines, following ethical approval and safety protocols from the Institutional Ethics Committee of a dental college in Chennai.

### 2.2 Sample size calculation

G∗Power 3.1.9.3 (for Mac OS X®️) to determine the sample size required for statistical significance. Based on previous studies investigating similar biomaterials, the calculation indicated a need for 12 samples per group to achieve a power of 0.80 with an alpha error of 0.05.^18^

### 2.3 Specimen preparation

A total of 24 samples were fabricated from two materials: titanium (N = 12, Coil, S-Tech Corp., Tainan City, Taiwan) and PEKK (N = 12, INTAM™ PEKK, Intamsys Technology Co. Ltd., Shanghai, China). The samples were fabricated in a disc shape as per the ISO standardization (ISO 10993-5), with a diameter of 10 mm and a thickness of 2 mm using STL files designed with Tinkercad software (Autodesk, San Francisco, CA). The samples were then processed using a 3D printer, ensuring uniformity across all specimens.

### 2.4 Cell cultures and chemicals

The Human Periodontal Ligament (PDL) fibroblast cell lines were sourced from the National Centre for Cell Science (NCCS), Pune, India. These cells were grown in Dulbecco's Modified Eagle Medium (DMEM; Caisson Laboratories, USA) enriched with 10 % fetal bovine serum (FBS; Gibco, Canada) and phosphate-buffered saline (PBS). The cultures were maintained at 37 °C in a humidified 5 % CO_2_ atmosphere, with 1 % antibiotic-antimycotic solution added to prevent contamination. For the experiments, JC-1 dye (5,5′,6,6′-tetrachloro-1,1′,3,3′-tetraethylbenzimidazolylcarbocyanine iodide) and a real-time PCR kit (CFX96 Touch™ system, Bio-Rad, USA) were procured from Invitrogen. All reagents used were of analytical grade and high purity.

### 2.5 Cytotoxicity assessment

**Fig. 1:**
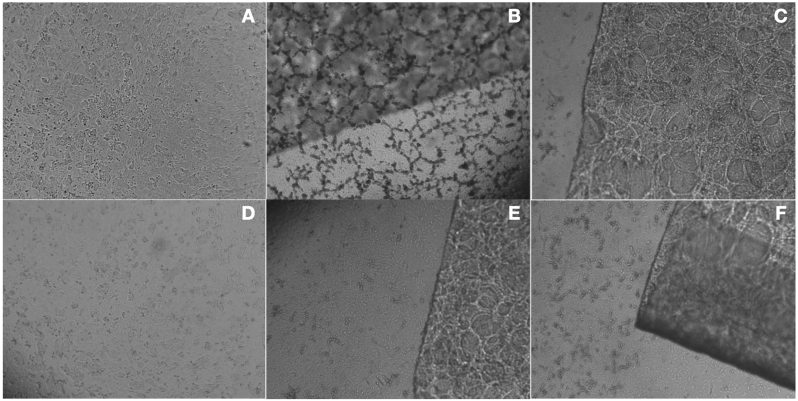
Assessment of cytotoxicity activity by exposure of human periodontal fibroblasts; A, B, C for 24 h duration, D, E, F for 48hrs duration for DMEM (control), titanium and PEKK respectively through MTT assay.

Human periodontal fibroblast (HPF) cells were seeded in 96-well plates at a density of 5 × 10^5^ cells/well and left to adhere overnight. Subsequently, triplicate cultures were exposed to experimental materials (titanium and PEKK) and incubated at 37 °C under 5 % CO_2_ for 24 and 48 h. After incubation, 3-(4,5-dimethylthiazol-2-yl)-2,5-diphenyltetrazolium bromide (MTT) reagent was added to each well, followed by an additional 3-h incubation at 37 °C. The resulting formazan crystals were solubilized with 200 μL dimethyl sulfoxide (DMSO), and absorbance was measured at 570 nm using a spectrophotometer. Untreated cells in DMEM served as the negative control for cytotoxicity assessment (Fig. 1).

The experiment was conducted in three independent replicates. Results are expressed as mean ± SD of optical density measurements. Cell viability (%) was calculated using the formula:

$$\%\;viability=\frac{OD\;extract\;treated}{OD\;negative\;control}\times 100$$

### 2.6 Cell culture and treatment

Human periodontal ligament fibroblasts (PDL cells) were plated in 6-well plates at a density of 2 × 10^4^ cells/well and allowed to attach overnight. The cells were then exposed to test materials (Titanium, PEKK, and a control group) in serum-free medium and incubated for 24 or 48 h at 37 °C under 5 % CO_2_.

### 2.7 RNA extraction and cDNA synthesis

After incubation, total RNA was isolated using the TRIzol® reagent (Ab Gene, UK) per the manufacturer's guidelines. RNA purity and concentration were assessed spectrophotometrically, with yields expressed in micrograms (μg). For cDNA synthesis, 2 μg of RNA was reverse-transcribed using a Eurogentec reverse transcriptase kit (Seraing, Belgium).

### 2.8 Quantitative real-time PCR (qPCR)

Gene expression analysis was done using Takara SYBR® Green Master Mix and gene-specific primers for IL-1β and TNF-α (sequences below). The PCR protocol included:

● Initial denaturation: 94 °C for 2 min
● Amplification: 35 cycles of 94 °C (1 min), 57 °C (30 s), 72 °C (30 s)
● Final extension: 72 °C for 10 min

### 2.9 Primer sequences

● IL-1β:
  ○ Forward: *CCACAGACCTTCCAGGAGAATG*
  ○ Reverse: *GTGCAGTTCAGTGATCGTACAGG*
● TNF-α:
  ○ Forward: *CTCTTCTGCCTGCTGCACTTTG*
  ○ Reverse: *ATGGGCTACAGGCTTGTCACTC*

Amplification specificity was verified via melting curve analysis, and β-actin served as the endogenous control. Reactions were run on a CFX96 Touch™ Real-Time PCR system (Bio-Rad, USA).

### 2.10 Data analysis

Relative gene expression was calculated using the 2^(−ΔΔCT) method, with normalization to β-actin. Fold changes in IL-1β and TNF-α expression were compared across treatment groups at 24 and 48 h. Statistical analysis was performed using CFX Manager™ Software (v2.1).

### 2.11 Molecular docking analysis

Molecular docking was used to explore the interactions between PEKK and biological molecules. The protein structures were obtained from experimental databases or predicted computationally. The 3D structure of PEKK was optimized, and docking was performed using AutoDock 1.5.6 software. The docking poses were ranked based on their binding affinity scores, and promising interactions were visually inspected using Biovia Discover Studio.

### 2.12 Statistical analysis

Prior to analysis, data distribution and variance homogeneity were verified using the Shapiro-Wilk normality test and Levene's test, respectively. Since both assumptions were met, parametric statistical methods were employed for further evaluation. Inter-group comparisons were performed using one-way analysis of variance (ANOVA) with Tukey's honestly significant difference (HSD) post hoc test for multiple comparisons, with statistical significance set at p < 0.05. All computations were executed using IBM SPSS Statistics software (Version 29.0; IBM Corp., Armonk, NY, USA).

## 3 Results

### 3.1 Cell cytotoxicity

At 24 h, PEKK demonstrated a mean cell viability of 79.58 ± 2.53 %, which was marginally higher than titanium at 78.08 ± 2.23 %. Similarly, at 48 h, PEKK exhibited 78.50 ± 2.81 % viability, compared to 76.92 ± 2.27 % for titanium (Table 1). While these differences suggest slightly better cytocompatibility for PEKK, the differences were not statistically significant (P > 0.05). Furthermore, pairwise comparisons using Tukey's post hoc test confirmed that while both PEKK and titanium significantly reduced cell viability compared to the control (P < 0.001), no significant difference was observed between PEKK and titanium at either 24 h (P = 0.160) or 48 h (P = 0.167, (Table 2). The optical microscope images revealed comparable HPF morphology and cellular density between each specific extract and the control suggesting that the materials are unlikely to have adverse effects on surrounding periodontal tissues**.** These findings indicate that both PEKK and titanium exhibit comparable cytocompatibility, with PEKK showing a trend toward slightly higher cell viability under the given experimental conditions. This is a crucial finding, suggesting that these materials, when used in dental or orthopaedic applications, do not induce significant cytotoxicity in HPFs.

**Table 1:** Assessment of cytotoxicity activity by indirect exposure of HPF cells to extracts of different implant materials through MTT assay using one way ANOVA test.

Time interval (hours)	Group	Mean ± SD (%)	Std. error	95 % CI		F value	P value
				lower	upper		
24 h	Control	100 ± 0	0	100	100	471.754	<0.001^a^
	Titanium	78.08 ± 2.23	0.64	76.66	79.50		
	PEKK	79.58 ± 2.53	0.73	77.97	81.19		
24 h	Control	100 ± 0	0	100	100	457.496	<0.001^a^
	Titanium	76.92 ± 2.27	0.66	75.47	78.36		
	PEKK	78.50 ± 2.81	0.81	76.71	80.28		

a The mean difference is significant at the 0.05 level.

**Table 2:** Pairwise comparison of cytotoxicity activity by indirect exposure of HPF cells to different implant materials through MTT assay usingPost hoc Tu-key test.

Time interval (hours)	Group	MD	Std. error	95 % CI		P value
				lower	upper	
24hrs	Control vs Titanium	21.91	0.79	19.96	23.87	0.001^a^
	Control vs PEKK	20.41	0.79	18.46	22.37	0.001^a^
	PEKK vs Titanium	1.50	0.79	−0.45	3.45	0.160
48hrs	Control vs Titanium	23.08	0.85	20.99	25.17	0.001^a^
	Control vs PEKK	21.50	0.85	19.41	23.59	0.001^a^
	PEKK vs Titanium	1.58	0.85	−0.51	3.67	0.167

a The mean difference is significant at the 0.05 level.

### 3.2 Gene expression analysis of pro-inflammatory cytokines

**Fig. 2:**
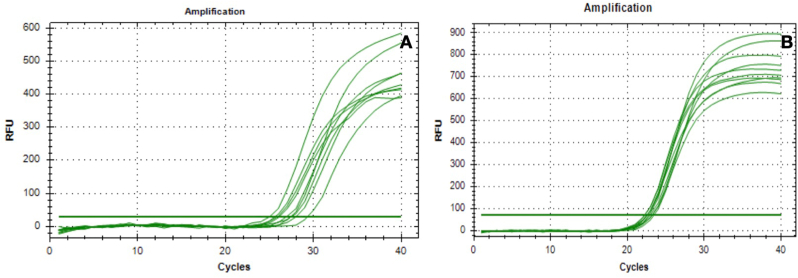
Gene expression of pro-inflammatory cytokines (a)TNF-α mRNA and (b) IL-1β mRNA in Human periodontal ligament fibroblasts cells after 24 h of cultivation in testing extracts.

After 24 h of culture with material extracts (Titanium and PEKK), human oral fibroblasts (HOF) showed differential expression of the pro-inflammatory cytokines IL-1β and TNF-α (Fig. 2). Quantitative analysis revealed statistically significant upregulation of both IL-1β (p < 0.01) and TNF-α (p < 0.05) mRNA levels in cells treated with LPS (positive control) compared to material-treated groups. Notably, no significant difference in inflammatory gene expression was observed between PEKK and titanium (p > 0.05), suggesting comparable biocompatibility in terms of inflammatory response (Fig. 2).

### 3.3 Molecular docking

**Fig. 3:**
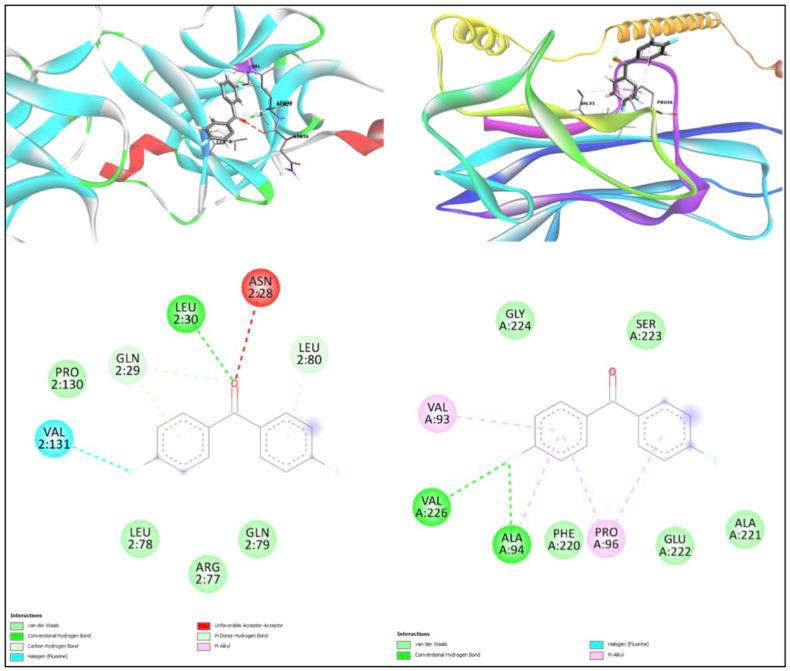
Molecular docking analysis results using PyRx software and 3D structure visualized using Biovia Discovery Studio.

Molecular docking was performed to evaluate the interaction of PEKK with key pro-inflammatory cytokines, IL-1β and TNF-α (Fig. 3). The docking results revealed that PEKK exhibited a binding energy of −8.9 kcal/mol with IL-1β, forming a hydrogen bond with the amino acid residue LEU30. For TNF-α, PEKK demonstrated a binding energy of −7.3 kcal/mol and established two hydrogen bonds with VAL226 and ALA94.

## 4 Discussion

The findings of this study indicate that both PEKK and titanium exhibit comparable cytocompatibility, with PEKK showing slightly higher cell viability under the given experimental conditions. Pro-inflammatory cytokine analysis demonstrated that the mRNA expression levels of IL-1β (P < 0.01) and TNF-α (P < 0.05) were significantly higher in the positive control (LPS) than in the PEKK and titanium groups, confirming the biocompatibility of these materials. PEKK and titanium demonstrated comparable effects (P > 0.05), suggesting that PEKK elicits a similar inflammatory response to titanium. Molecular docking analysis further highlighted PEKK's higher binding affinity for IL-1β (−8.9 kcal/mol) compared to TNF-α (−7.3 kcal/mol), indicating its potential to modulate inflammatory pathways and reduce inflammation. These findings collectively support the null hypothesis that PEKK and titanium exhibit similar biological responses, affirming PEKK's potential as an alternative implant material.

Several studies have explored the biocompatibility and mechanical properties of PEKK and titanium in biomedical applications.^19^, ^20^, ^21^ Titanium, known for its excellent osseointegration, has been the gold standard for decades. However, concerns such as allergic reactions, aesthetic limitations in anterior regions, and interference with MRI imaging have driven the search for alternatives.^22^, ^23^, ^24^ PEKK, a member of the polyaryletherketone (PAEK) family, has garnered attention due to its superior mechanical strength and biocompatibility. Alqurashi et al.^25^ emphasized PEKK's potential in implantology, highlighting its radiolucency, bone-like mechanical properties, and resistance to biofilm formation. Similarly, Rozeik et al.^26^ demonstrated comparable cell viability between PEKK and zirconia, but noted that PEKK's cytocompatibility improves significantly with surface treatments, such as sulfonation.

Our study aligns with these findings by showing comparable cell viability between PEKK and titanium. However, limited data exists on the inflammatory response induced by PEKK. Studies such as Peng et al.^27^ have reported that PEKK's pro-inflammatory cytokine expression is lower than that of unmodified PEEK, which is consistent with our results. Furthermore, molecular docking analyses in prior research have highlighted the importance of biomaterial interactions with cytokines such as IL-1β and TNF-α in determining biocompatibility.^28^ Our docking results are consistent with these findings, showing strong binding of PEKK to IL-1β, suggesting its potential to reduce localized inflammation. This contrasts with reports of higher inflammatory responses induced by unmodified PEEK, underscoring PEKK's advantages as an implant material.

Despite these promising results, discrepancies in the literature highlight areas requiring further exploration. For instance, zirconia, another alternative to titanium, exhibits excellent aesthetics but suffers from brittleness, limiting its use in load-bearing regions. Similarly, studies on PEKK have shown variability in cytocompatibility based on surface modifications, indicating that further optimization is necessary to maximize its clinical performance.^29^ Our study contributes to this growing body of evidence, offering insights into PEKK's biological performance and its potential to modulate inflammatory pathways.

Nevertheless, this study has limitations. This in vitro model has inherent limitations in simulating the complete physiological environment, particularly regarding immune cell interactions and systemic biological factors. Additionally, molecular docking is a computational approach and, while insightful, requires in vivo validation to confirm its findings. The scope of cytokine analysis was also limited, focusing on IL-1β and TNF-α, while other key inflammatory mediators, such as IL-6 and IL-10, were not evaluated. Future studies should explore these factors and include long-term in vivo assessments to provide a more comprehensive understanding of PEKK's performance.

## 5 Conclusion

This study demonstrated that PEKK exhibits comparable cytocompatibility and pro-inflammatory responses to titanium, with a trend toward slightly higher cell viability under experimental conditions. Molecular docking analysis revealed PEKK's strong and stable interaction with IL-1β, highlighting its potential to modulate inflammatory pathways. These findings support the null hypothesis that PEKK induces similar biological responses to titanium, reinforcing its viability as an alternative implant material. While the results are promising, further in vivo studies and long-term evaluations are necessary to validate PEKK's performance and establish its clinical applications in implantology.

## Patient consent

N/A.

## Ethical clearance

Not applicable.

## Source of funding

Nil.

## Declaration of competing interest

The authors declare the following financial interests/personal relationships which may be considered as potential competing interests:Subhabrata Maiti reports was provided by SIMATS Deemed University Saveetha Dental College. If there are other authors, they declare that they have no known competing financial interests or personal relationships that could have appeared to influence the work reported in this paper.
